# Gender Differences in Electronic Health Record Usage Among Surgeons

**DOI:** 10.1001/jamanetworkopen.2024.21717

**Published:** 2024-07-23

**Authors:** Karen Malacon, Gavin Touponse, Ezra Yoseph, Guan Li, Pingho (Janet) Wei, Kimberly Kicielinski, Lara Massie, Theresa Williamson, Summer Han, Corinna Zygourakis

**Affiliations:** 1 Department of Neurosurgery, Stanford University Medical Center, Stanford, California; 2 Stanford Health Care, Stanford, California; 3 Department of Neurosurgery, Medical University of South Carolina, Charleston; 4 Department of Neurosurgery, Allegheny Health Network, Monroeville, Pennsylvania; 5 Department of Neurosurgery, Massachusetts General Hospital, Harvard Medical School, Boston; 6 Quantitative Sciences Unit, Department of Medicine, Stanford University School of Medicine, Stanford, California

## Abstract

**Importance:**

Understanding gender differences in electronic health record (EHR) use among surgeons is crucial for addressing potential disparities in workload, compensation, and physician well-being.

**Objective:**

To investigate gender differences in EHR usage patterns.

**Design, Setting, and Participants:**

This cross-sectional study examined data from an EHR system (Epic Signal) at a single academic hospital from January to December 2022. Participants included 224 attending surgeons with patient encounters in the outpatient setting. Statistical analysis was performed from May 2023 to April 2024.

**Exposures:**

Surgeon’s gender.

**Main Outcomes and Measures:**

The primary outcome variables were progress note length, documentation length, time spent in medical records, and time spent documenting patient encounters. Continuous variables were summarized with median and IQR and assessed via the Kruskal-Wallis test. Categorical variables were summarized using proportion and frequency and compared using the χ^2^ test. Multivariate linear regression was used with primary EHR usage variables as dependent variables and surgeon characteristics as independent variables.

**Results:**

This study included 222 529 patient encounters by 224 attending surgeons, of whom 68 (30%) were female and 156 (70%) were male. The median (IQR) time in practice was 14.0 (7.8-24.3) years. Male surgeons had more median (IQR) appointments per month (78.3 [39.2-130.6] vs 57.8 [25.7-89.8]; *P* = .005) and completed more medical records per month compared with female surgeons (43.0 [21.8-103.9] vs 29.1 [15.9-48.1]; *P* = .006). While there was no difference in median (IQR) time spent in the EHR system per month (664.1 [301.0-1299.1] vs 635.0 [315.6-1192.0] minutes; *P* = .89), female surgeons spent more time logged into the system both outside of 7am to 7pm (36.4 [7.8-67.6] vs 14.1 [5.4-52.2] min/mo; *P* = .05) and outside of scheduled clinic hours (134.8 [58.9-310.1] vs 105.2 [40.8-214.3] min/mo; *P* = .05). Female surgeons spent more median (IQR) time per note (4.8 [2.6-7.1] vs 2.5 [0.9-4.2] minutes; *P* < .001) compared with male surgeons. Male surgeons had a higher number of median (IQR) days logged in per month (17.7 [13.8-21.3] vs 15.7 [10.7-19.7] days; *P* = .03). Female surgeons wrote longer median (IQR) inpatient progress notes (6025.1 [3692.1-7786.7] vs 4307.7 [2808.9-5868.4] characters/note; *P* = .001) and had increased outpatient document length (6321.1 [4079.9-7825.0] vs 4445.3 [2934.7-6176.7] characters/note; *P* < .001). Additionally, female surgeons wrote a higher fraction of the notes manually (17% vs 12%; *P* = .006). After using multivariable linear regression models, male gender was associated with reduced character length for both documentations (regression coefficient, −1106.9 [95% CI, −1981.5 to −232.3]; *P* = .01) and progress notes (regression coefficient, −1119.0 [95% CI, −1974.1 to −263.9]; *P* = .01). Male gender was positively associated with total hospital medical records completed (regression coefficient, 47.3 [95% CI, 28.3-66.3]; *P* < .001). There was no difference associated with gender for time spent in each note, time spent outside of 7 am to 7 pm, or time spent outside scheduled clinic hours.

**Conclusions and Relevance:**

This cross-sectional study of EHR data found that female surgeons spent more time documenting patient encounters, wrote longer notes, and spent more time in the EHR system compared with male surgeons. These findings have important implications for understanding the differential burdens faced by female surgeons, including potential contributions to burnout and payment disparities.

## Key Points

**Question:**

Are there gender differences in electronic health record (EHR) usage among surgeons?

**Findings:**

In this cross-sectional study of 224 attending surgeons, female surgeons spent more time logged into the EHR system outside of 7 am to 7 pm, spent more time in the EHR outside of scheduled clinic hours, spent more time per note, wrote longer progress and documentation notes, had fewer appointments, and wrote fewer medical records compared with their male colleagues.

**Meaning:**

This study’s results suggest the need for a comprehensive understanding of gender differences in EHR usage among surgeons and the associated implications.

## Teaser

This cross-sectional study examines gender differences in electronic health record (EHR) usage patterns among surgeons.

## Introduction

The widespread adoption of electronic health records (EHRs) has revolutionized health care delivery, transforming the way medical information is stored, accessed, and shared.^1^ However, as EHR usage becomes increasingly pervasive, concerns have arisen regarding its impact on health care professionals, including the potential for gender disparities in EHR utilization^2^ and associated consequences, such as physician burnout^3,4,5,6,7,8,9^ and amplification of the gender pay gap.^2^

There is a growing body of literature showing a gap in compensation between male and female surgeons, with male surgeons earning 36% more than female surgeons, and women in surgical subspecialties experiencing the largest difference in adjusted salary.^10,11,12^ Disparities in EHR use may represent uncompensated labor that is not accounted for in the already substantial gender pay gap.^2,13^ Given the mounting evidence of gender differences in EHR usage and the association between EHR-related tasks and burnout,^14^ it is imperative to investigate the variations in the burden of EHR usage among female vs male surgeons.

Prior studies on gender differences in EHR usage have consistently highlighted that female physicians spend more time in EHR documentation both during and after work hours compared with their male counterparts.^15,16,17^ However, these studies have largely focused on nonsurgical specialties, including primary care^15^ and physicians in ambulatory care .^17^ Due to the different practice patterns and day-to-day tasks of surgeons compared with nonsurgeons and the increasing proportion of female surgeons, there is a need to further study differences in EHR usage between male and female attending surgeons.

In light of these knowledge gaps, we conducted a comprehensive investigation into gender differences in EHR usage among surgeons. The primary objective of this study was to analyze EHR usage patterns among surgeons during the specified study period. We hypothesized that female surgeons face an increased burden in EHR usage compared with their male colleagues.

## Methods

### Study Design

This retrospective cross-sectional study used data obtained from an EHR system, Epic Signal (Epic Systems), from January 1, 2022, through December 31, 2022. Inclusion criteria were surgeons affiliated with a single academic institution, Stanford University, who had completed residency training and who saw any patients in the outpatient setting during the study period. This study was conducted in accordance with the Strengthening the Reporting of Observational Studies in Epidemiology (STROBE) reporting guideline.

### Ethical Considerations

As this study involved retrospective analysis of deidentified EHR data, it was exempted from the requirement for informed consent. Ethical approval for the study was obtained from the Stanford University institutional review board.

### Data Collection and Variables

We collected variables defining surgeon characteristics (gender, specialty, academic position, time in practice since residency), patient characteristics (age, items on problem list), and EHR variables (time in system, appointments, documentation characteristics, level of service [defined by *Current Procedural Terminology* billing codes for consults, established patients, or new patients levels 1-5], turnaround time, messaging/calls, orders). The EHR system dataset included gender information obtained from the physician’s personal information table and specialty data obtained from the department to which the physician belongs. Academic positions (affiliate, assistant professor, associate professor, professor, instructor) and time in practice were determined from the publicly available online Stanford Community Academic Profile (CAP) directory. Primary EHR variables were progress note length (inpatient note character length), documentation length (outpatient note character length), time spent in medical records, and time documenting patient encounters. The method of composition describes the percentage of notes written manually, using a SmartTool, transcription, or copy and paste. Time spent outside of scheduled clinic hours represents time spent logged into the EHR system 30 minutes or more before the first and after the last appointment scheduled, respectively, on clinic days (determined based on integration with the Cadence calendar system). Time spent outside 7am to 7pm is specific to scheduled clinic days. The time in system metric encompasses both clinic and nonclinic days. Inactive time, defined as periods when the Hyperspace window is not in focus or exceeds 5 seconds of inactivity, is not captured in the EHR system data.

### Statistical Analysis

We summarized EHR system data (ie, sum, average) across 1-month reporting periods for each clinician. The number of reporting periods varied depending on the time each surgeon worked over the year of data collection. To equally weigh each surgeon’s data within the analysis despite varying time worked, all variables were averaged across reporting periods for each surgeon to yield a single number for each variable per surgeon. Continuous variables were summarized with median and IQR. Median and IQR were used to report variables with nonnormal distributions which were assessed via the Kolmogorov-Smirnov test and visually with histograms (eFigure in Supplement 1). The Kruskal-Wallis test was used to compare nonnormal continuous variables between male and female surgeons. Categorical variables were summarized using proportion and frequency and compared using the χ^2^ test. Histograms and scatterplots were used to visualize data. Outlying data points were removed from scatterplots using the Robust regression and OUTlier removal package in GraphPad Prism 8 (GraphPad Software) with *Q* of 0.1% (most conservative) to better visualize the distributions. Outlier removal was not conducted for mean and median values with accompanying *P* values in data tables.

Multivariate linear regression was used with primary EHR usage variables as dependent variables (documentation length, progress note length, time in notes per note, total hospital medical records, time outside 7am to 7pm, time outside scheduled clinic hours) and surgeon gender, time in practice since residency, surgeon specialty, surgeon academic appointment, average patient age, and average number of problem list items as independent variables. To address potential multicollinearity in our model, we implemented a variance inflation factor (VIF) cutoff of 4, which resulted in the removal of the variable *academic appointment* from the analysis. Several specialties were grouped together to address potential unstable numeric issues during model fitting, stemming from sparse cell counts present in several specialties. Specifically, for the regression analysis, surgeon specialties were grouped into the following categories: cardiothoracic surgery, neurosurgery, orthopedic surgery, plastic and hand surgery, general surgery (including general surgery, colon and rectal surgery, transplant surgery, endocrine surgery, and trauma surgery), otolaryngology, podiatry, urology, and vascular surgery. Two-sided *P* ≤ .05 were considered statistically significant. Statistical analysis was conducted using R programming language version 4.1 (R Project for Statistical Computing) from May 2023 to April 2024.

## Results

We identified 222 539 patient encounters by 224 surgeons affiliated with a single academic institution, of whom 68 (30%) were female and 156 (70%) were male (eTable 1 in Supplement 1). This included 171 812 progress notes, 252 883 documents, and a total of 2 181 819 minutes in the EHR in our analysis. The median (IQR) time in practice was 14.0 (7.8-24.3) years. Surgeons spanned 14 specialties, with the most common being general surgeons (n = 45 [20%]) and orthopedic surgeons (n = 45 [20%]) (eTable 1 in Supplement 1).

The orthopedic surgery department had the highest proportion of male surgeons (84%), compared with general surgery which had a higher proportion of female surgeons (51%) (eTable 2 in Supplement 1). Male surgeons represented a higher proportion of full professors (38%), whereas female surgeons comprised a higher proportion of assistant professors (46%) (Table 1). Male surgeons had longer median (IQR) time in practice compared with female surgeons (17.0 [9.0-27.0] years vs 9.0 [5.0-18.3] years; *P* < .001).

**Table 1.** Clinician Characteristics Stratified by Gender

Characteristic	Surgeons, No. (%)		
	Female (n = 68)	Male (n = 156)	*P* value
Specialty			
Cardiothoracic surgery	8 (11.8)	12 (7.7)	.004
Colon and rectal surgery	0	2 (1.3)	
Endocrine surgery	1 (1.5)	1 (0.6)	
General surgery	23 (33.8)	22 (14.1)	
Hand surgery	3 (4.4)	0	
Neurological surgery	4 (5.9)	16 (10.3)	
Orthopedic surgery	7 (10.3)	38 (24.4)	
Otolaryngology	10 (14.7)	27 (17.3)	
Plastic surgery	2 (2.9)	10 (6.4)	
Podiatry	1 (1.5)	3 (1.9)	
Transplant surgery	1 (1.5)	0	
Trauma surgery	0	3 (1.9)	
Urology	4 (5.9)	16 (10.3)	
Vascular surgery	4 (5.9)	6 (3.8)	
Academic appointment			
Affiliate	3 (4.4)	13 (8.3)	.01
Assistant professor	31 (45.6)	35 (22.4)	
Associate professor	13 (19.1)	44 (28.2)	
Professor	19 (27.9)	59 (37.8)	
Instructor	2 (2.9)	5 (3.2)	
Time in practice, median (IQR), y	9.0 (5.0-18.3)	17.0 (9.0-27.0)	<.001

**Figure 1.**
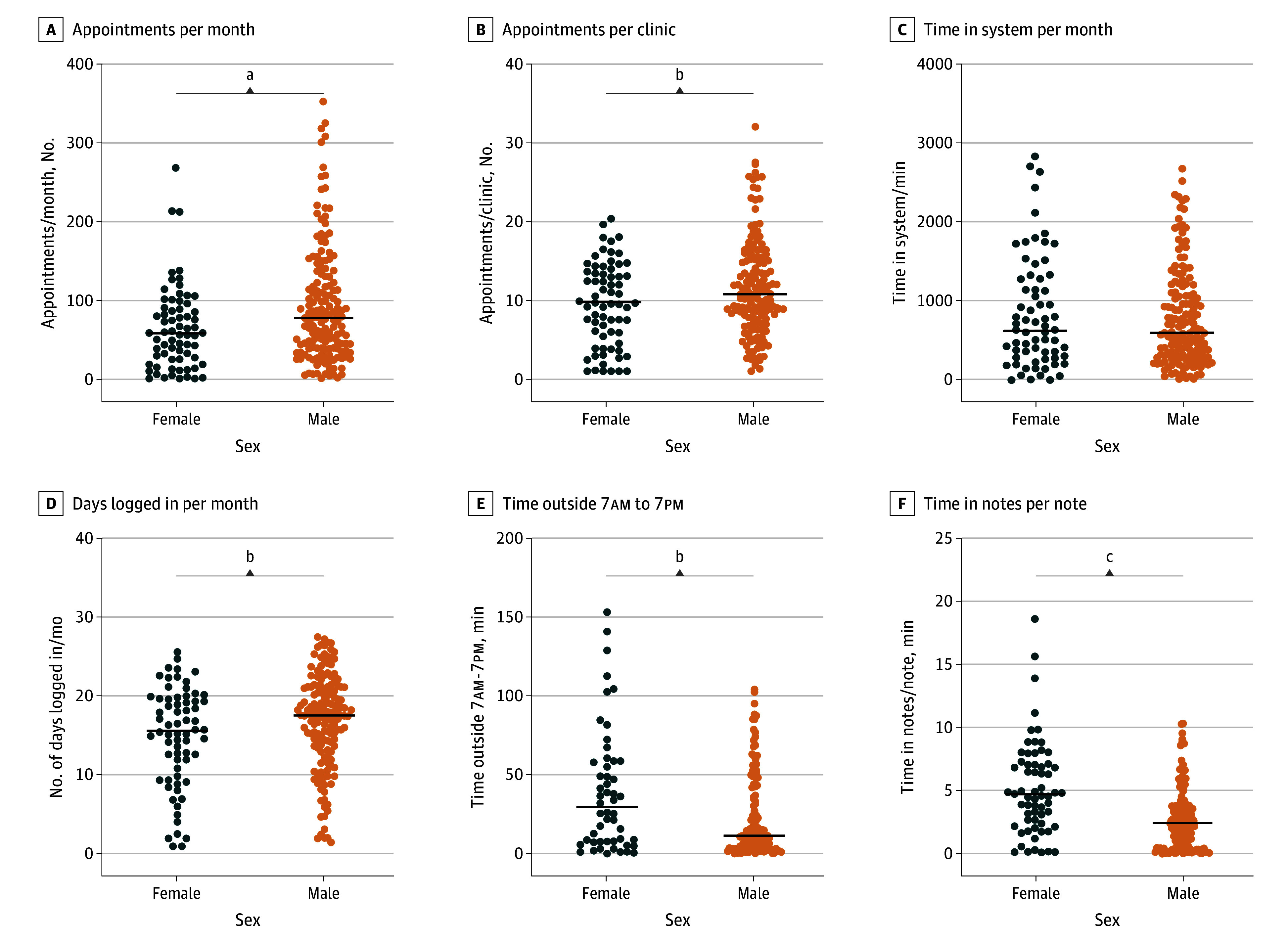
Scatterplots Demonstrate Individual Data Points and Median Values for Clinic Schedule and EHR Usage Variables Stratified by Gender ^a^*P* ≤ .01. ^b^*P* ≤ .05. ^c^*P* ≤ .001.

Patients treated by female and male surgeons were similar in median [IQR] age (58.8 [54.0-63.4] years vs 57.6 [53.2-63.7] years; *P* > .99) and had a similar number of problem list items (9.6 [8.0-11.4] items vs 9.1 [7.9-10.4] items; *P* = .15) (Table 2). On average, male surgeons had more median (IQR) appointments per month (78.3 [39.2-130.6] vs 57.8 [25.7-89.8]; *P* = .005) and had more appointments per clinic day (10.8 [7.6-15.3] vs 9.6 [5.2-13.5]; *P* = .05) (Figure 1).

**Table 2.** Patient Characteristics, Clinic Schedule, and EHR Usage Among Surgeons Stratified by Gender

Variable	Patient, clinic, and EHR usage, median (IQR)		
	Female (n = 68)	Male (n = 156)	*P* value
Patient characteristics			
Patient age, y	58.8 (54.0-63.4)	57.6 (53.2-63.7)	>.99
Items on problem list	9.6 (8.0-11.4)	9.1 (7.9-10.4)	.15
Clinic schedule			
No. of appointments per month	57.8 (25.7-89.8)	78.3 (39.2-130.6)	.005
Appointments per clinic day	9.6 (5.2-13.5)	10.8 (7.6-15.3)	.05
EHR usage			
Time in system per month, min	664.1 (301.0-1299.1)	635.0 (315.6-1192.0)	.89
Time in notes per note, min	4.8 (2.6-7.1)	2.5 (0.9-4.2)	<.001
No. of days logged in per month, d	15.7 (10.7-19.7)	17.7 (13.8-21.3)	.03
Time outside scheduled clinic hours per month, min	134.8 (58.9-310.1)	105.2 (40.8-214.3)	.05
Time outside of 7am-7pm per month, min	36.4 (7.8-67.6)	14.1 (5.4-52.2)	.05
Medical record characteristics			
Medical records completed per month	29.1 (15.9-48.1)	43.0 (21.8-103.9)	.006
Time to complete hospital medical records, days	1.8 (0.8-2.8)	1.9 (0.9-3.2)	.72
Progress notes written per month	43.6 (13.8-78.1)	45.6 (18.0-102.3)	.33
Progress note length, character	6025.1 (3692.1-7786.7)	4307.7 (2808.9-5868.4)	.001
Documents written per month	66.2 (23.1-128.6)	68.2 (28.6-150.9)	.40
Document length, characters	6321.1 (4079.9-7825.0)	4445.3 (2934.7-6176.7)	<.001
Fraction of notes written by clinician (vs other)	0.6 (0.3-0.9)	0.6 (0.2-1.0)	.50
Fraction of notes written by other (vs practitioner)	0.4 (0.1-0.7)	0.4 (0.0-0.8)	.50
Method of medical record composition			
% Written manually	17.2 (9.4-33.6)	11.6 (1.5-24.6)	.01
% Written using SmartTool	48.0 (28.5-61.3)	46.9 (20.3-64.3)	.92
% Written using transcription	0	0.0 (0.0-13.5)	.08
% Using copy/paste	9.7 (2.5-22.2)	5.2 (0.1-15.6)	.04
Level of service (*CPT* code)			
% Consult level 1 (99 241)	0.0	0.1 (0.6)	.49
% Consult level 2 (99 242)	6.6 (16.4)	8.4 (21.4)	.75
% Consult level 3 (99 243)	15.2 (19.6)	32.6 (34.8)	.05
% Consult level 4 (99 244)	39.4 (32.9)	41.2 (36.1)	.86
% Consult level 5 (99 245)	38.9 (41.9)	17.8 (27.6)	.02
% Established patient level 1 (99 211)	0.1 (0.6)	0.1 (0.6)	.85
% Established patient level 2 (99 212)	6.8 (16.4)	9.9 (15.9)	.20
% Established patient level 3 (99 213)	37.7 (24.2)	42.0 (27.4)	.28
% Established patient level 4 (99 214)	40.2 (22.5)	36.5 (26.1)	.32
% Established patient level 5 (99 215)	15.1 (18.2)	11.5 (18.3)	.19
% New patient level 1 (99 201)	0.0	0.0	NaN
% New patient level 2 (99 202)	1.8 (4.7)	4.2 (11.2)	.10
% New patient level 3 (99 203)	18.1 (22.1)	32.7 (30.0)	.001
% New patient level 4 (99 204)	41.7 (28.9)	40.6 (29.1)	.80
% New patient level 5 (99 205)	38.4 (34.2)	22.6 (31.9)	.001

Abbreviations: *CPT*, *Current Procedural Terminology*; EHR, electronic health record; NaN, not a number.

There was no significant difference in median (IQR) time spent in the EHR system per month (664.1 [301.0-1299.1] vs 635.0 [315.6-1192.0] minutes; *P* = .89); however, male surgeons had a higher number of days logged in per month (17.7 [13.8-21.3] vs 15.7 [10.7-19.7] days; *P* = .03). Female surgeons spent more time logged into the system outside of scheduled clinic hours per month (134.8 [58.9-310.1] vs 105.2 [40.8-214.3] minutes; *P* = .05) and outside of 7am to 7pm per month (36.4 [7.8-67.6] vs 14.1 [5.4-52.2] minutes; *P* = .05). Female surgeons spent more time per note (4.8 [2.6-7.1] vs 2.5 [0.9-4.2] minutes; *P* < .001) compared with male surgeons.

**Figure 2.**
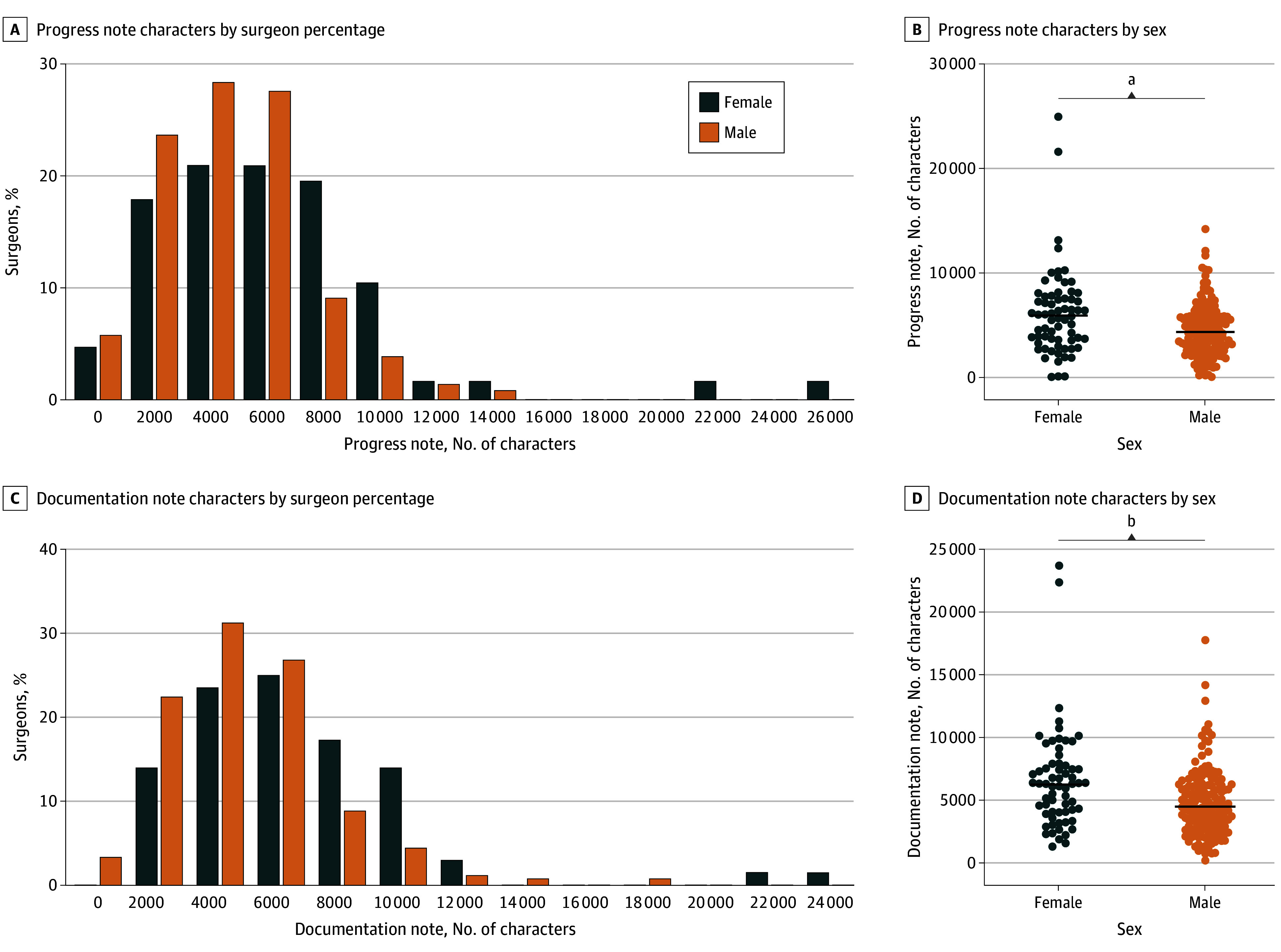
Distributions and Scatterplots of Progress Note and Documentation Length Stratified by Gender ^a^*P* ≤ .01. ^b^*P* ≤ .001.

Male surgeons completed more median (IQR) medical records per month compared with female surgeons (43.0 [21.8-103.9] vs 29.1 [15.9-48.1]; *P* = .006) (Table 2). Median (IQR) time to complete hospital medical records (1.8 [0.8-2.8] vs 1.9 [0.9-3.2] days; *P* = .72), median (IQR) number of progress notes written per month (43.6 [13.8-78.1] vs 45.6 [18.0-102.3]; *P* = .33), and median (IQR) number of documents written per month (66.2 [23.1-128.6] vs 68.2 [28.6-150.9]; *P* = .40) were not statistically different between male and female surgeons. Female surgeons wrote longer progress notes (6025.1 [3692.1-7786.7] vs 4307.7 [2808.9-5868.4] characters; *P* = .001) and had increased document length (6321.1 [4079.9-7825.0] vs 4445.3 [2934.7-6176.7] characters; *P* < .001) (Figure 2). While there was no significant difference in fraction of notes written by surgeon vs other or using transcription or SmartTools, female surgeons wrote a higher fraction of the notes manually (17% vs 12%; *P* = .006) and a higher fraction using copy and paste (10% vs 5%; *P* = .04). Female surgeons had a higher percentage of level 5 consult visits (38.9% vs 17.8%; *P* = .02), a higher percentage of level 5 new patient visits (38.4% vs 22.6%; *P* = .001), a lower percentage of level 3 consult visits (15.2% vs 32.6%; *P* = .05), and a lower percentage of level 3 new patient visits (18.1% vs 32.7%; *P* = .001), as compared with their male colleagues. There were no significant differences between female and male surgeons in turnaround time, messages and/or calls, and appointments closed per month (eTable 3 in Supplement 1).

Multivariable linear regression analyses were performed to determine the relative association of gender with primary EHR variables (Table 3). After controlling for surgeon’s years in practice, specialty, median patient age, and median number of problem list items, surgeon’s male gender was associated with 1107 fewer characters for documentation length (regression coefficient, −1106.9 [95% CI, −1981.5 to −232.3]; *P* = .01) and 1119 fewer characters for progress note length (regression coefficient, −1119.0 [95% CI, −1974.1 to −263.9]; *P* = .01). Gender was not significantly associated with time spent in each note (regression coefficient, −1.1 [95% CI, −3.7 to 1.5]; *P* = .40), time spent outside of 7am to 7pm per month (regression coefficient, −19.8 [95% CI, −55.5 to 15.9]; *P* = .28), or time spent outside scheduled clinic hours (regression coefficient, −54.8 [95% CI, −119.7 to 10.1]; *P* = .10) (eTable 4 in Supplement 1). Male gender was positively associated with completing 47 more total hospital medical records per month (regression coefficient, 47.3 [95% CI, 28.3-66.3]; *P* < .001). Cardiothoracic surgery was associated with significantly shorter documentation length; orthopedic surgery, plastic and hand surgery, and podiatry were all associated with significantly shorter documentation length and progress note length as compared with general surgery (all *P* < .05) (Table 3). Cardiothoracic surgery was associated with more hospital medical records completed per month (*P* < .001), while orthopedic surgery, otolaryngology, plastic and hand surgery, and urology were associated with fewer completed hospital medical records per month than general surgery (all *P* < .02) (Table 3). Despite lower medical record completion, otolaryngology and urology were associated with significantly higher time outside 7am to 7pm and scheduled clinic hours (all *P* < .01) (eTable 4 in Supplement 1).

**Table 3.** Multivariable Linear Regression Models for Documentation Length, Progress Note Length, and Total Hospital Medical Records Completed

Variable	Documentation length		Progress note length		Number of medical records completed/mo	
	Regression coefficient (95% CI)	*P* value	Regression coefficient (95% CI)	*P* value	Regression coefficient (95% CI)	*P* value
Surgeon gender						
Female	[Reference]	.01	[Reference]	.01	[Reference]	<.001
Male	−1106.9 (−1981.5 to −232.3)		−1119.0 (−1974.1 to −263.9)		47.3 (28.3 to 66.3)	
Years practicing since residency	−26.2 (−62.5 to 10.1)	.16	−16.6 (−52.1 to 18.8)	.36	−0.5 (−1.4 to 0.3)	.23
Specialty						
General surgery^a^	[Reference]	NA	[Reference]	NA	[Reference]	NA
Cardiothoracic surgery	−1624.5 (−3204.3 to −44.7)	.05	−1335.7 (−2937.8 to 266.4)	.10	63.1 (29.0 to 97.3)	<.001
Neurosurgery	−1382.7 (−2910.3 to 144.9)	.08	−1033.8 (−2527.6 to 460.0)	.18	32.8 (−0.5 to 66.1)	.06
Orthopedic surgery	−3485.4 (−4645.3 to −2325.6)	<.001	−3682.4 (−4819.6 to −2545.3)	<.001	−40.8 (−66.4 to −15.3)	.002
Otolaryngology	−451.0 (−1684.4 to 782.4)	.47	−709.9 (−1919.1 to 499.3)	.25	−65.9 (−94.4 to −37.3)	<.001
Plastic and hand surgery	−2908.8 (−4581.0 to −1236.5)	<.001	−3265.3 (−4895.8 to −1634.8)	<.001	−44.9 (−82.7 to −7.1)	.02
Podiatry	−4104.2 (−7065.3 to −1143.1)	.007	−4101.1 (−6985.1 to −1217.1)	.006	−72.6 (−145.2 to 0.1).	.05
Urology	−679.0 (−2226.6 to 868.6)	.39	−886.5 (−2396.4 to 623.3)	.25	−71.4 (−104.9 to −37.9)	<.001
Vascular surgery	−3720.0 (−5832.0 to −1608.0)	<.001	−3503.4 (−5559.9 to −1447.0)	<.001	4.1 (−42.9 to 51.1)	.86
Median patient age	17.8 (−43.8 to 79.4)	.57	4.7 (−55.3 to 64.7)	.88	1.3 (−0.05 to 2.6).	.06
Median No. of problem list items	58.1 (−141.9 to 258.1)	.57	69.5 (−126.1 to 265.1)	.49	−2.0 (−6.8 to 2.8)	.42

Abbreviation: NA, not applicable.

^a^ General surgery reference category includes general surgery, colon and rectal surgery, transplant surgery, endocrine surgery, and trauma surgery.

## Discussion

In this study, we report gender differences in EHR usage between male and female surgeons at a large academic hospital. Specifically, we observed that despite seeing 74% of patients and completing 67% of medical records per month compared with their male counterparts, female surgeons spent an equivalent amount of time in the EHR system. Furthermore, female surgeons spent 28% more time in the system outside of scheduled clinic hours and 158% more time outside of 7am to 7pm, despite seeing fewer patients and completing fewer hospital medical records. In addition, female surgeons spent more time writing individual notes compared with their male colleagues. Finally, after controlling for years in practice, specialty, average patient age, and average number of items in patient problem list in multivariate linear regression, female gender was significantly associated with fewer total hospital medical records completed per month and greater progress note and average documentation note length.

These findings are consistent with previous studies showing gender disparities in EHR usage among health care professionals. Several studies report that women physicians in nonsurgical specialties spend more overall time in the EHR per day, more time doing this work during nonscheduled hours, and more time handling in-basket messages.^15,16,17^ Our study adds to this body of evidence by demonstrating that these disparities exist within the surgical field as well. While prior studies shed light on the substantial time surgical residents dedicate to EHR usage,^18,19,20^ they did not examine EHR use among attending surgeons or provide gender-specific stratification. While we found that female and male surgeons spent an equal amount of total time in the EHR system, female surgeons had on average 21 fewer appointments per month, suggesting that time spent per patient on EHR was greater for female vs male surgeons. Female surgeons also spent more time in the EHR system after hours, which may contribute to greater burnout.^21^

The reasons behind the differences in patient volume and medical record writing between male and female surgeons warrant further exploration. It is possible that variations in career choices and preferences may contribute to these disparities. For instance, women surgeons may opt for lighter schedules to balance personal and professional responsibilities,^22^ leading to a lower patient load. Additionally, the administrative burden associated with EHR usage may influence patient volume, as female surgeons spend substantial time on documentation tasks, leaving less time available for patient care. Patients also may have differing expectations of male vs female physicians, expecting more time and communication with female physicians.^23,24,25^ These practices could exacerbate the gender pay gap^2^ and/or contribute to increased burnout in female surgeons.^3,26^ We also report differences in documentation length across various specialties, specifically with orthopedic surgeons, plastic and hand surgeons, and podiatrists writing shorter notes. This is not surprising, given these specialties are often focused on one specific problem that may be more straightforward than those in other surgical specialties. Other studies have reported similar differences in note length among surgical specialties,^27^ which may stem from heterogeneous documentation standards, templates, or strategies (eg, transcription use), in addition to complexity of cases and patients.^15^

In our study, female surgeons wrote inpatient progress notes that were 40% longer and outpatient documentations that were 42% longer than their male colleagues. Furthermore, female surgeons manually documented their notes 42% more frequently compared with their male counterparts. Administrative burdens that disproportionately fall on female surgeons, such as the expectation of more comprehensive documentation, could potentially devalue the work performed by female surgeons. Furthermore, although we could not differentiate whether the clinical notes were cosigned by advanced practice practitioners (APPs), it is important to highlight that additional APP support frequently depends on productivity and clinic volume. If the presence of APPs is proven to enhance productivity, it might unintentionally contribute to a self-reinforcing cycle, potentially exacerbating existing gender disparities.

We found that female surgeons had 2 times the percentage of level 5 consult and new patient visits compared with their male colleagues. Level 5 billing is the highest level of complexity for new patient visits and is typically used when the patient’s condition requires a comprehensive evaluation and management.^28,29^ Given the increased time spent by female surgeons in the EHR, it may be appropriate that female surgeons are billing more frequently for a higher level of service. However, we are unable to determine whether the increased relative value units from the higher level of service visits would counteract the effect of the fewer patients seen overall by female vs male surgeons when determining overall productivity and pay. Our findings are consistent with existing literature showing that female surgeons invest more time in individual patient encounters.^30^ This discovery further supports the widespread criticism that productivity metrics based solely on volume do not adequately capture the true extent of physician work.^31,32^

Addressing these disparities requires a multifaceted approach. Policy interventions aimed at promoting equity in compensation could include adjusting payment structures to recognize the additional time and effort invested in EHR usage by female surgeons. Moreover, reassessing the documentation requirements and teaching efficient documentation practices may help alleviate the administrative burden on all surgeons while maintaining the necessary quality of care. Implementing targeted EHR concierge services specifically for women surgeons could alleviate documentation-related stress and enhance overall efficiency. At our institution, EHR concierge services are currently offered equally, and voluntarily, to all physicians. These services include personalized needs assessment, professional observation, and individualized training, all of which have demonstrated substantial uptake and positive outcomes.^33^ Providing individual physician feedback on EHR use patterns may encourage clinicians to improve through the use of smart tools, templates, and preference lists to enhance efficiency.^17^ Future research should investigate the complex interplay between gender expectations, patient outcomes, and the time allocated to clinical encounters. Recognizing and addressing the gender disparities in workload is crucial for promoting diversity, equity, and inclusion within the surgical field.

### Limitations

This study has limitations. It was conducted at a single academic hospital, potentially limiting the generalizability of our findings to other settings or populations. Furthermore, this study relied on the accuracy and completeness of the EHR data recorded in the EHR system, which introduces the possibility of errors or omissions within the data. Furthermore, we acknowledge that the observed gender differences may not reflect intrinsic differences in EHR usage between males and females but rather reflect that EHR systems may have been created by and beta-tested on a greater proportion of male clinicians and thus may be more user-friendly to this group. Moreover, it is important to acknowledge that comparing clinician documentation across specialties may be challenging due to variations in practice patterns and documentation requirements. While we report data on the proportion of the notes that is written by the surgeon themselves, we do not have granularity on who is contributing to the remainder of the note. Specifically, we do not know how different team members such as residents, fellows, scribes, nurses, or APPs contribute to the percentage of the documentation that is not completed by the surgeon themselves. There may be gender differences in access to these various resources depending on seniority and specialty. Despite these limitations, this study contributes to the existing EHR utilization literature and highlights the need for further policy interventions to optimize EHR workflows and mitigate potential burdens associated with EHR usage in surgical practice.

## Conclusions

This study found gender differences in EHR usage among surgeons, underscoring the importance of policy changes to address compensation disparities, alleviate potential administrative burdens, and provide targeted support to female surgeons. By fostering a more equitable and supportive environment, we can enhance the well-being and success of women in surgical careers while improving patient outcomes.

## Notes

Supplement 1. **eFigure.** Histogram of Years Practicing Since Residency Showing Nonnormal Distribution **eTable 1.** Summary for All Practitioner Characteristics **eTable 2.** Practitioner Characteristics by Specialty and Academic Appointment **eTable 3.** EHR Messaging, Turnaround, Appointment Closure and Billing Characteristics Stratified by Gender **eTable 4.** Multivariable Linear Regression Models for Time in Notes per Note, Time Outside 7am-7pm/mo to Min, and Time Outside of Scheduled Hours/mo to Min

Supplement 2.
Data Sharing Statement
