# Supplementary material for: Gender Differences in Electronic Health Record Usage Among Surgeons
Source: JAMA Netw Open. 2024 Jul 23;7(7):e2421717. doi: 10.1001/jamanetworkopen.2024.21717 (PMC11267410; doi:10.1001/jamanetworkopen.2024.21717)
Supplement: Supplement 1. — eFigure. Histogram of Years Practicing Since Residency Showing Nonnormal Distribution eTable 1. Summary for All Practitioner Characteristics eTable 2. Practitioner Characteristics by Specialty and Academic Appointment eTable 3. EHR Messaging, Turnaround, Appointment Closure and Billing Characteristics Stratified by Gender eTable 4. Multivariable Linear Regression Models for Time in Notes per Note, Time Outside 7am-7pm/mo to Min, and Time Outside of Scheduled Hours/mo to Min [file jamanetwopen-e2421717-s001.pdf]

Supplemental Online Content

Malacon K, Touponse G, Yoseph E, et al. Gender differences in electronic health record usage among surgeons. *JAMA Netw Open.* 2024;7(7):e2421717. doi:10.1001/jamanetworkopen.2024.21717

eFigure. Histogram of Years Practicing Since Residency Showing Non-Normal Distribution

eTable 1. Summary for All Practitioner Characteristics

eTable 2. Practitioner Characteristics by Specialty and Academic Appointment

eTable 3. EHR Messaging, Turnaround, Appointment Closure and Billing Characteristics Stratified by Gender

eTable 4. Multivariable Linear Regression Models for Time in Notes per Note, Time Outside 7am-pm/mo to Min, and Time Outside of Scheduled Hours/mo to Min

This supplemental material has been provided by the authors to give readers additional information about their work.

© 2024 Malacon K et al. *JAMA Network Open.*

eFigure. Histogram of Years Practicing Since Residency Showing Non-Normal Distribution

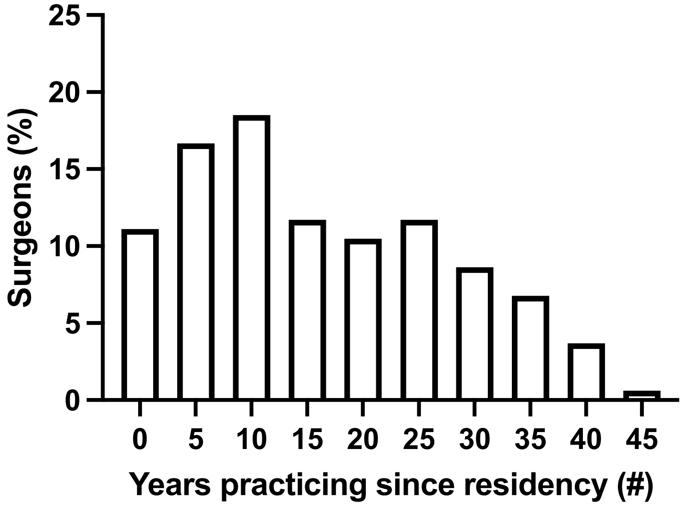

© 2024 Malacon K et al. *JAMA Network Open.*

eTable 1. Summary for All Practitioner Characteristics

	N=224
Gender, n (%)	
Female	68 (30.4)
Male	156 (69.6)
Time in practice, years, median [IQR]	14.0 [7.8, 24.3]
Specialty, n (%)	
Cardiothoracic Surgery	20 (8.9)
Colon and Rectal Surgery	2 (0.9)
Endocrine Surgery	2 (0.9)
General Surgery	45 (20.1)
Hand Surgery	3 (1.3)
Neurological Surgery	20 (8.9)
Orthopedic Surgery	45 (20.1)
Otolaryngology	37 (16.5)
Plastic Surgery	12 (5.4)
Podiatry	4 (1.8)
Transplant Surgery	1 (0.4)
Trauma Surgery	3 (1.3)
Urology	20 (8.9)
Vascular Surgery	10 (4.5)
Academic appointment, n (%)	
Affiliate	16 (7.1)
Assistant Professor	66 (29.5)
Associate Professor	57 (25.4)
Professor	78 (34.8)
Other	7 (3.1)
Outpatient encounters, n	222,529
Progress notes, n	171,812
Documents, n	252,883
Time in system, min	2,181,819

© 2024 Malacon K et al. *JAMA Network Open.*

eTable 2. Practitioner Characteristics by Specialty and Academic Appointment

Characteristic	No. (%) of Surgeons		
	**Female (n = 68)**	**Male (n = 156)**	**P-value**
Specialty			
Cardiothoracic Surgery	8 (40)	12 (60)	0.3201
Colon and Rectal Surgery	0 (0)	2 (100)	>0.9999
Endocrine Surgery	1 (50)	1 (50)	0.5159
General Surgery	23 (51)	22 (49)	0.0011
Hand Surgery	3 (100)	0 (0)	0.0271
Neurological Surgery	4 (20)	16 (80)	0.4446
Orthopedic Surgery	7 (16)	38 (84)	0.0179
Otolaryngology	10 (27)	27 (73)	0.6992
Plastic Surgery	2 (17)	10 (83)	0.3545
Podiatry	1 (25)	3 (75)	>0.9999
Transplant Surgery	1 (100)	0 (0)	0.3036
Trauma Surgery	0 (0)	3 (100)	0.5552
Urology	4 (20)	16 (80)	0.4446
Vascular Surgery	4 (40)	6 (60)	0.4962
Academic appointment			
Affiliate	3 (19)	13 (81)	0.4024
Assistant Professor	31 (47)	35 (53)	0.0008
Associate Professor	13 (23)	44 (77)	0.1828
Professor	19 (24)	59 (76)	0.1719
Instructor	2 (29)	5 (71)	>0.9999

© 2024 Malacon K et al. *JAMA Network Open.*

eTable 3. EHR Messaging, Turnaround, Appointment Closure and Billing Characteristics Stratified by Gender

	EHR messaging, turnaround, and appointment characteristics, median (IQR)		
	Female (n = 68)	Male (n = 156)	P value
**Turnaround time**			
Staff messages received per month	38.3 [13.8, 72.7]	35.1 [14.8, 69.9]	0.98
Time to mark staff message as done, min	13.9 [4.7, 61.0]	7.2 [2.0, 52.3]	0.06
**Messages/calls**			
Patient calls per month	17.0 [12.7, 20.5]	18.5 [14.7, 22.0]	0.09
Patient medical requests per month	16.8 [12.8, 20.2]	18.2 [14.3, 21.8]	0.10
In basket messages received per day from staff	2.0 [1.1, 3.4]	1.6 [0.8, 3.0]	0.34
In basket messages received per day from patients	0.1 [0.1, 0.4]	0.1 [0.1, 0.3]	0.72
In basket messages received per day for patient medical requests	0.5 [0.2, 1.5]	0.7 [0.2, 1.9]	0.50
**Appointments closed**			
Fraction appointments closed same day	0.55 [0.36, 0.85]	0.63 [0.28, 0.93]	0.52
Fraction appointments closed 1-2 days	0.11 [0.03, 0.22]	0.09 [0.02, 0.21]	0.86
Fraction appointments closed 2-3 days	0.04 [0.01, 0.12]	0.03 [0.00, 0.08]	0.39
Fraction appointments closed 4-5 days	0.01 [0.00, 0.054]	0.02 [0.00, 0.08]	0.36
Fraction appointments closed 7+ days	0.05 [0.00, 0.22]	0.03 [0.00, 0.21]	0.45
**Orders**			
Fraction orders with team contributions completed by provider	0.63 [0.29, 0.91]	0.53 [0.20, 0.96]	0.61

© 2024 Malacon K et al. *JAMA Network Open.*

eTable 4. Multivariable Linear Regression Models for Time in Notes per Note, Time Outside 7am-pm/mo to Min, and Time Outside of Scheduled Hours/mo to Min

	**Time in notes per note**		**Time outside 7am-7pm/month (min)**		**Time outside of scheduled hours /month (min)**	
**Variable**	**Regression Coefficient (95% CI)**	**P value**	**Regression Coefficient (95% CI)**	**P value**	**Regression Coefficient (95% CI)**	**P value**
**Provider gender**						
Female (ref)	-		-		-	
Male	-1.1 (-3.7, 1.5)	0.40	-19.8 (-55.5, 15.9)	0.28	-54.8 (-119.7, 10.1)	0.10
Years practicing since residency	-0.2 (-0.3, -0.04)	0.01**	0.4 (-1.1, 1.9)	0.61	1.3 (-1.4, 4.0)	0.35
**Specialty**						
General surgery◊ (ref)	-		-	-	-	
Cardiothoracic surgery	-1.2 (-5.8, 3.4)	0.61	7.1 (-57.5, 71.7)	0.83	-34.7 (-167.1, 97.6)	0.61
Neurosurgery	-1.7 (-6.1, 2.8)	0.47	35.1 (-29.1, 99.2)	0.29	8.6 (-107.1, 124.3)	0.88
Orthopedic surgery	-3.4 (-6.7, 0.03).	0.05*	6.6 (-40.8, 54.0)	0.79	-9.5 (-94.1, 75.1)	0.83
Otolaryngology	0.8 (-2.8, 4.4)	0.67	77.3 (27.2, 127.4)	0.003**	171.4 (80.0, 262.8)	<0.001***
Plastic and hand surgery	-2.8 (-7.6, 2.1)	0.27	-8.4 (-78.9, 62.0)	0.81	-62.4 (-185.2, 60.4)	0.32
Podiatry	-3.2 (-11.8, 5.5)	0.47	-35.0 (-151.5, 81.5)	0.56	45.5 (-163.8, 254.8)	0.67
Urology	-0.9 (-5.4, 3.60)	0.69	82.8 (20.1, 145.5)	0.01**	146.9 (33.1, 260.7)	0.01**
Vascular surgery	-3.7 (-9.8, 2.5)	0.25	7.9 (-79.3, 95.1)	0.86	-88.6 (-244.9, 67.7)	0.27
Average patient age	-0.01 (-0.2, 0.2)	0.95	-2.0 (-4.7, 0.8)	0.16	-0.2 (-5.2, 4.8)	0.94
Average number of problem list items	0.02 (-0.7, 0.6)	0.95	7.3 (-1.2, 15.7)	0.09	10.0 (-5.5, 25.5)	0.21

© 2024 Malacon K et al. *JAMA Network Open.*
