# Supplementary material for: Gender Differences in Electronic Health Record Usage Among Surgeons
Source: JAMA Netw Open. 2024 Jul 23;7(7):e2421717. doi: 10.1001/jamanetworkopen.2024.21717 (PMC11267410; doi:10.1001/jamanetworkopen.2024.21717)
Supplement: Supplement 2. — Data Sharing Statement [file jamanetwopen-e2421717-s002.pdf]

Data Sharing Statement

Malacon. Gender Differences in Electronic Health Record Usage Among Surgeons. *JAMA Netw Open*. Published July 23, 2024. doi:10.1001/jamanetworkopen.2024.21717

Data
Data available: No

Additional Information
Explanation for why data not available: The participants of this study did not give written consent for their data to be shared publicly, so due to the sensitive nature of the research supporting data is not available.
